# Unique Predictors of Sleep Quality in Junior Athletes: The Protective Function of Mental Resilience, and the Detrimental Impact of Sex, Worry and Perceived Stress

**DOI:** 10.3389/fpsyg.2019.01256

**Published:** 2019-05-31

**Authors:** Maria Hrozanova, Frode Moen, Ståle Pallesen

**Affiliations:** ^1^Department of Neuromedicine and Movement Science, Faculty of Medicine and Health Sciences, Norwegian University of Science and Technology, Trondheim, Norway; ^2^Department of Education and Lifelong Learning, Faculty of Social an Educational Sciences, Norwegian University of Science and Technology, Trondheim, Norway; ^3^Department of Psychosocial Science, Faculty of Psychology, University of Bergen, Bergen, Norway

**Keywords:** sleep, athletes, stress, worry, sex, resilience

## Abstract

Since athletic development and functioning are heavily dependent on sufficient recuperation, sleep in athletes is becoming a topic of increasing interest. Still, existing scientific evidence points to inadequate sleep in athletes, especially in females. This may be due to the fact that sleep is vulnerable to disturbances caused by stress and cognitive and emotional reactions to stress, such as worry and negative affect, which may exacerbate and prolong the stress response. Such disturbing factors are frequently experienced by junior athletes aiming for performance development and rise in the rankings, but may be damaging to athletic progression. Based on limited research in non-athletic samples, mental resilience may protect individuals against the detrimental effects of stress on sleep. Therefore, the present study aimed to investigate the extent to which sex, mental resilience, emotional (negative affect) and cognitive (worry) reactions to stress, and perceived stress, uniquely contributed to sleep quality in a cross-sectional study including 632 junior athletes. A multiple hierarchical linear regression showed that females had poorer sleep quality than males, while the mental resilience sub-components Social Resources and Structured Style were positively associated with sleep quality, providing a protective function and thus preventing sleep quality from deteriorating. Simultaneously, worry, as well as perceived stress, were negatively associated with sleep quality. Together, the independent variables explained 28% of the variance in sleep quality. A dominance analysis showed that perceived stress had the largest relative relationship with sleep quality. Based on these results, close attention should be paid to athletes’ abilities to manage worry and perceived stress, and the potential of mental resilience as a protective factor that could prevent sleep from deteriorating. The latter might be especially relevant for female athletes. Since performance margins are progressively becoming smaller and smaller, every improvement that adequate sleep can provide will be beneficial in terms of improved functioning and athletic performance.

## Introduction

Sleep is essential for the progression toward athletic excellence. The scheduling, quantity and quality of sleep has been highlighted as decisive for athletes’ ability to train, improve performance, prevent injury and recover (Mah et al., 2011; Simpson et al., 2017). In line with principal physiological and psychological recovery strategies, sleep is crucial for optimal functioning of all major systems of the body (Zee et al., 2014). However, the sleep of aspiring athletes may be far from optimal (for a review, see Gupta et al., 2017), possibly due to the heavy psycho-physiological stress loads inherent to athleticism (Hausswirth et al., 2014).

Indeed, previous research has shown that late evening exercise, training regimes with inadequate recovery time, excessive training, as well as long travels across time zones, all detrimentally effect the sleep and performance of athletes (reviewed in O’Donnell et al., 2018a). This might in particular be true for female athletes, who may experience poorer sleep quality than male athletes (Swinbourne et al., 2016; Hoshikawa et al., 2018). Previous research has determined that without a balance between psycho-physiological stress loads and recovery, progression toward elite sport performance might be at risk (Kellmann et al., 2018). However, it has to be emphasized that most of the research on stress loads and recovery in athletes has concerned physiological stress loads. Factors such as total training load, endurance training and strength training undoubtedly play a role in the inadequate sleep of athletes, by inducing longer sleep latencies, more awakenings, non-refreshing sleep and daytime fatigue (Gupta et al., 2017). It is striking, however, that despite the importance of the psychological demands athletes continuously have to face as they develop their performance, there is a dearth of research on the role of psychological variables, and how these relate to sleep quality.

In order to appreciate the role of heightened psycho-physiological stress loads in athletic recovery, it is necessary to understand how psychological stress and reactions to stress influence recuperation in athletic settings. The stress response is activated when an athlete is faced with an acute stressor. A cascade of important physiological and behavioral changes is triggered, mobilizing resources that aid the athlete in overcoming the stressor (Chrousos and Gold, 1992). Furthermore, upon the occurrence of the stressor, the athlete immediately engages in appraisal of situational demands (primary appraisal) and appraisal of the available resources for coping (secondary appraisal) with the stressor (Lazarus and Folkman, 1984). This context-specific appraisal is thought to determine the outcome of the stress reaction (Blascovich et al., 2001). Importantly, when athletes perceive that they lack resources to manage the situations they are up against, a maladaptive stress response may be triggered (Lazarus, 1974; Reme et al., 2008). Although currently understudied, appraisals during a maladaptive stress response are assumed to trigger emotional and cognitive reactions to stress, which may preserve, extend and exacerbate the stress response (Brosschot et al., 2006; Leger et al., 2018), contributing to an imbalance between psycho-physiological stress loads and recovery in athletes, and thus contributing to poor sleep quality.

The emotional and cognitive reactions to stress which occur during a maladaptive stress response may take many forms, but among the commonly occurring are emotional reactions that involve negative affect (Watson et al., 1988b), and cognitive reactions such as worrying (Borkovec et al., 1998). Worrying is thought to encompass repetitive, uncontrollable thoughts focusing on potential adversities of the future (Roemer and Borkovec, 1993), while negative affect is the tendency to experience a broad range of negative mood states, such as anxiety, fear, hostility, sadness, and loneliness (Watson et al., 1988a). Indeed, cognitive and emotional reactions to stress have previously been identified as major contributing factors to sleep disturbances (Harvey, 2002; Baglioni et al., 2010), likely caused by difficulties repressing mental activity while attempting to sleep (Harvey, 2000). Therefore, athletes experiencing negative cognitive and emotional reactions to stress may be at risk of disturbed sleep. Previous studies have shown that many athletes suffer from poor sleep, which among other factors, may lead to negative stress (Biggins et al., 2018). Few studies have investigated the directionality between stress and sleep. In one longitudinal study of PhD students, day-level stress was not directly related to sleep variables. However, day-level stress was positively associated with perseverative cognitions (worry) which were significantly related to impaired sleep (Van Laethem et al., 2016). In a study of Dutch employees, it was also found that worry mediated the relationship between stress and sleep, but the study also found that poor sleep quality was related to an increase in work-related stress reported over time (Van Laethem et al., 2015). Other studies have shown that poor sleep predicts stress levels (Wu et al., 2015) and that stress predicts poor sleep (Akerstedt et al., 2012). Taken together, these studies attest to a bi-directionality between stress and sleep that may proceed into a vicious cycle.

Importantly, not all individuals exposed to stressors experience disturbed sleep. In fact, some individuals have the ability to overcome adversity and adapt to new circumstances in a positive way, despite the triggering event itself being negative (Fitzpatrick, 2010). This ability is thought to be due to high mental resilience. Mental resilience is defined as a multifaceted phenomenon (Cicchetti and Garmezy, 1993), with the capacity to safeguard against the development of psychopathology in times of adversity (Hjemdal et al., 2006a). Friborg et al. (2003) have specified that the concept of mental resilience is “not only referring to important psychological skills or abilities, but also to the individual’s ability to use family, social and external support systems to cope better with stress.” Previous research suggests that the elite athlete population generally is characterized by unique resilient competencies (Fletcher and Sarkar, 2012). In fact, the high-performing elite athlete population is not passively and randomly exposed to challenges. On the contrary, athletes actively seek to engage in demanding situations on a continuous basis, in order to improve their performance level and boost competitiveness. The capacity to perceive stressors as opportunities for growth is based on positive appraisals relating to the psycho-physiological stressors athletes are up against. Importantly, in individuals who are able to overcome and adapt to adversity, mental resilience may provide a protective function in terms of sleep quality in both adults and adolescents (Notario-Pacheco et al., 2011; Brand et al., 2016).

The aim of the current study was to implement a psychological perspective on sleep quality in athletes, by investigating the extent to which sex, mental resilience, negative affect, worry and perceived stress, uniquely contribute to sleep quality in a large sample of junior athletes. This research provides a conceptual framework for investigating and understanding why some junior athletes obtain good sleep despite their excessive exposure to psycho-physiological stress loads. Four hypotheses were investigated. First, it was hypothesized that females experience poorer sleep quality than men (H1). Second, it was hypothesized that mental resilience is positively associated with sleep quality (H2). Third, it was hypothesized that negative affect and worry, variables denoting emotional and cognitive reactions to stress, are negatively associated with sleep quality (H3). Finally, perceived stress was hypothesized to be negatively associated with sleep quality (H4).

## Materials and Methods

### Participants

Participants were recruited from Norwegian high schools specialized for elite sports. Students at all high schools specialized for elite sports in Norway were invited to participate. A list of names and e-mail addresses was obtained via cooperation between the Center for Elite Sports Research and Olympic Top Sport Center, a national Norwegian organization that is part of Norwegian Olympic and Paralympic Committee and Confederation of Sports, with responsibility for training and management of elite athletes. A total sample of 1917 athletes were invited to participate.

Junior athletes comprise athletes up to the age of 20 years. Norwegian high schools specialized for elite sports, from which the current sample was drawn, have both elite and non-elite athlete students. Elite athletes are characterized as athletes who qualify for national teams, athletes who are members of a recruiting squad for that team at the time of this study, or athletes who perform at the highest level in national competitions, aspiring to become members of the representing national team. Non-elite athletes denotes athletes competing at the middle or lower level of national competitions.

### Design and Sample Size Estimation

In order to investigate the relationships between sex, resilience, negative affect, worry, subjective stress, and sleep quality, a cross-sectional design was utilized. Data was collected at one specific point in time with the use of a survey emailed to all participating athletes.

An *a priori* power analysis, using the G^∗^Power 3.1.9.4 (Faul et al., 2009), was performed for sample size estimation for the regression analysis. Setting the effect size to small/medium (*f*^2^ = –0.05), alpha to 0.05, power (1 – β) to 0.80, including 9 predictors, showed that 322 subjects would be needed to detect that *R*^2^ significantly deviated from zero.

### Ethics Statement

Prior to the start of the study, all participants provided informed written consent. Since all participants were 16 years or older, parental consent was not necessary. The Regional Committee for Medical and Health Research Ethics (REC) in Central Norway approved the study (document ID 725589).

### Instruments

Prior to the beginning of the study, the authors had to decide on the most appropriate measures. In terms of assessment of resilience, both a relevant scale for adults (Friborg et al., 2005) as well as for adolescents (Hjemdal et al., 2006a) has been developed. As the target sample comprised young adults typically living away from home, performing on a high level, and not dependent on their parents in their day-to-day life, and based on advice from the authors of the two aforementioned scales, it was decided to use the Resilience Scale for Adults (Friborg et al., 2005).

In terms of sleep measures the value of objective measures such as polysomnography or actigraphy, is indisputable. However, due to the relatively large sample size of the present study, its cross-sectional design, and the limited capacity of the participants to engage in more comprehensive data collection procedures, the use of such instruments as well as sleep diaries kept over time was deemed unsuitable. Therefore, the widely used, valid and reliable Pittsburgh Sleep Quality Index (Buysse et al., 1989) was chosen as measurement of sleep quality. Factors such as mindfulness (Lau et al., 2018) and self-compassion (Hu et al., 2018), which both may relate to sleep, were not assessed in the present study as other factors (e.g., resilience) that may counteract stress, were included. The decision to keep the questionnaire as short as possible was also based on research showing an inverse relationship between questionnaire length and response rate (Galesic and Bosnjak, 2009).

The survey included questions about demographics (age, sex), type of sport, performance level, motivations and ambitions. Standard questionnaires included the Resilience Scale for Adults (RSA), the Positive and Negative Affect Schedule (PANAS), the Penn State Worry Questionnaire (PSWQ), the Perceived Stress Scale (PSS), and the Pittsburgh Sleep Quality Index (PSQI). The survey took approximately 15 to 20 min to complete, and athletes had 1 month to submit their responses. One reminder was sent to athletes that did not respond.

#### Resilience Scale for Adults (RSA)

The RSA (Friborg et al., 2005) is a 33-item comprehensive measure of protective factors related to mental resilience in adults; the ability to cope with stress and negative experiences. Each item is scored on a seven-point Likert scale, ranging from 1 to 7, with varying options based on the context of the question. The questionnaire taps into six factors, including (1) Perception of the Self (e.g., “My assessments and decisions,” rated from 1 “I often doubt,” to 7 “I rely fully on”), (2) Planned Future (e.g., “My goals for the future are,” rated from 1 “Unclear” to “Well thought-through”), (3) Social Competence (e.g., “Coming up with good conversation topics is,” rated from 1 “Difficult” to 7 “Easy”), (4) Family Cohesion (e.g., “In my family, we like to,” rated from 1 “Find activities we can do together” to 7 “Do things separately”), (5) Social Resources (e.g., “My close friends/family members,” rated from 1 “Value my traits” to 7 “Do not like my traits”), and (6) Structured Style (e.g., “Rules and regular routines,” rated on 1 “Are missing from my everyday” to 7 “Are a part of my everyday”). Each of these factors measures different aspects of resilience (Hjemdal et al., 2006b), thus supporting the theoretical conceptualization of resilience as a multidimensional phenomenon (Cicchetti and Garmezy, 1993). Higher scores indicate stronger resilience. The scale has been shown to be valid, reliable, stable and satisfactorily operationalized (Friborg et al., 2003; Hjemdal et al., 2006b). Cronbach’s alphas were 0.79, 0.77, 0.76, 0.79, 0.81, and 0.63 for the aforementioned subscales, respectively.

#### The Positive and Negative Affect Schedule (PANAS)

The PANAS (Watson et al., 1988b) consists of two subscales that measure positive (PA) and negative emotions (NA). Each of the subscales contains 10 items. The PA scale reflects affects such as being inspired, strong, and enthusiastic; while the NA scale contains items reflecting affects such as afraid, distressed, and hostile. On a Likert scale from 1 (not at all) to 5 (very much), athletes rated the extent to which they experienced each specific affect within the last week. The factor structure of the PANAS has previously been supported in a study among young athletes (Crocker, 1997). The scale was shown to be reliable, valid and efficient for the assessment of positive and negative affect (Watson et al., 1988b) and Cronbach’s alphas in the current study were 0.87 and 0.86 for PA and NA, respectively.

#### Penn State Worry Questionnaire (PSWQ)

The PSWQ consists of 16 items investigating the propensity to engage in worry (e.g., “Many situations make me worry”). Each item is rated on a five-point Likert scale ranging from 1 (not at all typical) to 5 (very typical). Higher scores indicate higher worry. A validated Norwegian version of the PSWQ (Meyer et al., 1990; Pallesen et al., 2006) that has shown high reliability and validity, in line with the original PSWQ (Davey, 1993; Molina and Borkovec, 1994; Pallesen et al., 2006) was used. The Cronbach’s alpha for the PSWQ in the present study was 0.93.

#### Perceived Stress Scale (PSS)

The PSS (Cohen et al., 1983) measures self-appraised stress (e.g., “During the past month, how often have you felt that you were unable to control the important things in your life?”) on a 5-point Likert-type scale from 0 (never) to 4 (very often). More specifically, the PSS measures the degree to which respondents find their lives unpredictable, uncontrollable, and overloading (Cohen et al., 1983). The questions are general in nature and relatively context-free (Cohen and Williamson, 1988). Higher scores indicate more stress. The scores may be categorized into three stress levels: scores 0–13 indicate low stress, scores 14–26 indicate moderate stress, and scores 27–40 indicate high stress. The Norwegian version of the PSS, translated by Alfheim et al. (2012) was used. The PSS has been shown to have acceptable psychometric properties (Lee, 2012). The Cronbach’s alpha for the PSS measurement was 0.84.

#### Pittsburgh Sleep Quality Index (PSQI)

The PSQI (Buysse et al., 1989) is a 19-item questionnaire that discriminates between good and poor sleepers by measuring subjective sleep quality and sleep disturbances. The items cover a broad range of factors associated with sleep quality and sleep disturbances over the past month, including habitual sleep patterns (e.g., “How long (in minutes) has it taken you to fall asleep each night?”), the subjective impact of sleep on daytime functioning (e.g., “How often have you had trouble staying awake while driving, eating meals, or engaging in social activity?”), and the frequency and severity of perceived sleep problems (e.g., “How often have you had trouble sleeping because you cough or snore loudly?”). PSQI utilizes a combination of free entry answers and 4-point Likert scale options. The composite PSQI score ranges from 0 to 21, and a cutoff of 5 has been used to categorize participants into good sleepers (≤5) and poor sleepers (>5) (Buysse et al., 1989). In the present study, the Norwegian version of PSQI, which has been shown to possess good psychometric properties, was utilized (Pallesen et al., 2005). Cronbach’s alpha for the PSQI, when treating each of the components as items, was 0.74.

### Statistical Analyses

Preliminary analyses were conducted to ensure no violation of the assumption of normality, linearity, multicollinearity and homoscedasticity (see Supplementary Material). Descriptive statistics, the first-order partial Spearman correlation coefficient and two multiple linear regressions were conducted using IBM SPSS (version 25.0). In addition, an *a priori* and a *post hoc* power analysis were conducted using the G^∗^Power software package (Faul et al., 2009).

Composite scores for each of the included questionnaires and their respective subscales were calculated according to their relevant scoring manuals. These results are presented as mean ± SD. Furthermore, the first-order partial Spearman correlation coefficient was calculated to estimate the linear relationships between the investigated variables, controlling for gender.

To investigate the effects of multiple explanatory variables (predictors) on subjective sleep quality, multiple linear regression models were applied. The independent variables included sex, the six components of resilience (Perception of the Self, Planned Future, Social Competence, Family Cohesion, Social Resources, and Structured Style), negative affect and worry, and perceived stress. Due to restricted age range in the sample, age was not included as a predictor. The outcome variable was the composite PSQI score determining sleep quality.

**FIGURE 1:**
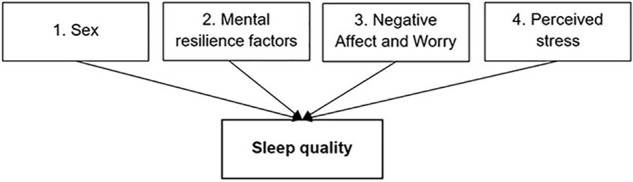
The input order of the investigated independent variables into the regression analysis, with the dependent variable marked in bold.

Two multiple linear regressions were carried out. In the first model, all of the predictor variables were entered in the same step, and the output was examined to see which of the individual predictors contributed significantly to the model’s ability to predict subjective sleep quality. In the second model, only the predictors that were significant at alpha level of *p* < 0.05 were entered into a hierarchical multiple linear regression, in order to identify the unique contribution of each individual predictor. Variables were entered in a pre-determined order: sex, as the control variable, was entered in Step 1. Since the mental resilience factors are considered to be stable characteristics, these were entered in the next steps, in order to identify the unique contribution of each individual mental resilience factor to sleep quality. Negative affect and worry were entered in Step 3, and perceived stress in Step 4, as cognitive and emotional reactions to stress has been suggested to preserve, extend and exacerbate perceived stress in athletes (see Figure 1). Statistical significance for all tests was set at alpha level of *p* < 0.05.

Finally, since it was expected that a moderate degree of correlation between some of the predictors tested in the multiple regression models will be present, dominance analysis was carried out in order to determine the relative importance of the individual predictor variables on the dependent variable in this study. Dominance analysis is based on estimating an *R*^2^ value for all possible subset models, as outlined in Azen and Budescu (2003). Even though this statistical analysis is especially useful in instances when not enough evidence is available to establish a specific theory regarding the importance of predictors (Johnson and LeBreton, 2004), dominance analysis can also be used in hierarchical models to confirm or dispute the theory’s predictions relating to the predictors’ order of input (Azen and Budescu, 2003). The dominance analysis was calculated using the DOMIN add-on module (Luchman, 2013) in Stata (version 15.0).

## Results

### Demographics

In all, 1917 athletes were invited to participate in the online survey of whom 670 completed the survey, providing a response rate of 34.9%. 38 participants were excluded from the analyses due to missing values on the PSQI. Therefore, the final sample included 632 participants. Of these, 50.2% were men, and 49.8% were women. Mean age was 18 ± 0.9, range 16–20 years. Of all athletes, 38.6% were defined as elite athletes, while 61.4% were defined as non-elite athletes. As many as 78.2% of all athletes had ambitions to become future elite athletes, while 21.8% did not have this ambition.

### Sleep Patterns

Results of the PSQI showed that on average, athletes spent 8:26 ± 0:56 h in bed, achieving 7:46 ± 00:59 h of sleep per night. On average, athletes fell asleep at 22:32 ± 00:49, woke up at 06:46 ± 00:38, and mean sleep onset latency was 22 ± 17 min. The mean composite PSQI score was 4.9 ± 3.0. Applying the cutoff value of 5, 69.8% of all athletes were defined as good sleepers, while 30.2% were poor sleepers.

### Correlations and Descriptive Statistics

The investigated variables included the subscales of the RSA (variables 1–6), negative affect of the PANAS (variable 7), and the sum scores of the PSWQ (variable 8), PSS (variable 9) and PSQI (variable 10). Table 1 shows the results of bivariate Pearson product-moment correlation coefficients of the investigated variables, as well as statistical means, standard deviations, and minimum and maximum scores.

**Table 1:** First-order partial correlation coefficients between the investigated variables and descriptive statistics, when controlling for gender, based on cross-sectional data collected from 632 junior athletes.

Variable	1	2	3	4	5	6	7	8	9	10
1. Perception of Self	–	0.60	0.40	0.44	0.51	0.40	−0.39	−0.56	−0.63	−0.35
2. Planned Future		–	0.29	0.41	0.46	0.45	−0.32	−0.34	−0.50	−0.29
3. Social Competence			–	0.32	0.41	0.22	−0.14	−0.25	−0.25	−0.13
4. Family Cohesion				–	0.66	0.34	−0.24	−0.27	−0.38	−0.29
5. Social Resources					–	0.37	−0.33	−0.31	−0.44	−0.32
6. Structured Style						–	−0.20	−0.17	−0.34	−0.28
7. Negative affect							–	0.44	0.57	0.35
8. Worry								–	0.64	0.37
9. Perceived stress									–	0.42
10. Sleep quality										–
										
Mean	4.9	5.2	5.1	5.8	6.1	5.1	22.8	44.8	24.5	4.9
SD	1.2	1.3	1.1	1.0	0.9	1.2	7.2	13.8	7.7	3.0
Minimum	1.0	1.0	1.3	1.3	2.1	1.0	10.0	16.0	4.0	0.0
Maximum	7.0	7.0	7.0	7.0	7.0	7.0	50.0	80.0	52.0	18.0

*All Spearman correlations are significant at the 0.00 level. N = 632.*

### Hierarchical Multiple Linear Regression

Two multiple linear regressions were carried out to investigate the extent to which the independent variables could uniquely predict subjective sleep quality (see Figure 1 to see the specific entry order). The first model (results shown in Supplementary Material) indicated that sex, two RSA subscales (Social Resources and Structured Style), worry and perceived stress significantly predicted sleep quality. In the second model, these significant predictors were each entered in separate blocks to identify the unique contribution of each individual predictor (see Table 2).

**Table 2:** Summary of hierarchical multiple regression analysis for variables predicting subjective sleep quality, based on cross-sectional data collected from 632 junior athletes.

	B	SE B	β	*R*^2^	Δ*R*^2^
**Model 1**					
Sex (♂ = 0, ♀ = 1)	0.84	0.24	0.14^∗^	0.02	0.02
					
**Model 2**					
Sex (♂ = 0, ♀ = 1)	1.03	0.22	0.17^∗^		
Social Resources	−1.23	0.13	−0.35^∗^	0.14	0.12
					
**Model 3**					
Sex (♂ = 0, ♀ = 1)	1.22	0.22	0.20^∗^		
Social Resources	−0.96	0.13	−0.28^∗^		
Structured Style	−0.58	0.10	−0.23^∗^	0.19	0.05
					
**Model 4**					
Sex (♂ = 0, ♀ = 1)	0.63	0.22	0.11^∗∗^		
Social Resources	−0.70	0.13	−0.20^∗^		
Structured Style	−0.51	0.10	−0.21^∗^		
Worry	0.06	0.01	0.29^∗^	0.26	0.07
					
**Model 5**					
Sex (♂ = 0, ♀ = 1)	0.51	0.22	0.08^∗∗∗^		
Social Resources	−0.53	0.14	−0.15^∗^		
Structured Style	−0.41	0.10	−0.16^∗^		
Worry	0.04	0.01	0.18^∗^		
Perceived stress	0.08	0.01	0.21^∗^	0.28	0.02

*^∗^p < 0.000, ^∗∗^p < 0.01, ^∗∗∗^p < 0.05.*

### *Post hoc* Power Calculation

A *post hoc* power calculation (Faul et al., 2009) for achieved power (*R*^2^ deviated from zero) was also performed. The achieved *R*^2^ of 0.28 equals an effect size (*f*^2^) of 0.39 (Cohen, 1988). The alpha level of 0.05 and *N* = 632 suggest a power of 1.00.

### Dominance Analysis

A dominance analysis was carried out in order to determine the relative importance of the predictor variables included in the original multiple linear regression model. The dominance analysis showed that perceived stress (rank 1) had the largest relative relationship with sleep quality, followed by worry (rank 2), the mental resilience factor Perception of Self (rank 3), negative affect (rank 4), the mental resilience factors Structured Style (rank 5), Social Resources (rank 6), Planned Future (rank 7), Family Cohesion (rank 8), Sex (rank 9) and finally, the mental resilience factors Social Competence (rank 10). Results of the dominance analysis are presented in Table 3.

**Table 3:** Results from dominance analysis showing dominance statistics and rank of relationships with sleep quality, based on cross-sectional data collected from 632 junior athletes.

Variable	Dominance statistics^a^	Standardized dominance statistics	Rank
Sex	0.01	0.03	9
Perception Of Self	0.03	0.12	3
Planned Future	0.03	0.10	7
Social Competence	0.01	0.02	10
Family Cohesion	0.02	0.01	8
Social Resources	0.03	0.10	6
Structured Style	0.03	0.10	5
Negative affect	0.03	0.11	4
Worry	0.05	0.16	2
Perceived stress	0.06	0.21	1

*^a^Refers to the contribution to prediction based on the squared semipartial correlation.*

## Discussion

The present study aimed to investigate the extent to which sex, mental resilience, emotional and cognitive reactions to stress, and perceived stress, uniquely contribute to sleep quality in a large sample of junior athletes. In support of H1, female sex predicted worse sleep quality. Partly supporting H2, results showed that the mental resilience sub-components Social Resources and Structured Style were positively associated with sleep quality, providing a protective function and thus preventing sleep quality from deteriorating. H3 was only partially supported – no support was found for the role of negative affect, but worry was negatively associated with sleep quality. Finally, perceived stress was negatively associated with sleep quality, which lend support to H4. The analyses in the current study revealed noteworthy results, which will now be discussed in light of existing research.

In line with previous research, the present study showed that female sex uniquely predicted significant proportions (2.0%) of the variance in poor sleep quality (Swinbourne et al., 2016; Hoshikawa et al., 2018). In the study by Hoshikawa et al. (2018), keeping a regular sleep/wake schedule, not thinking about troubles in bed, and depressive mood were significant predictors of poor sleep quality in females. One of the reasons why the female sex may have contributed to poorer sleep quality is that women in general experience greater prevalence of perceived stress (Matud, 2004) and worry (Zlomke and Hahn, 2010). Importantly, research is yet to systematically evaluate sex differences in the sleep quality of junior athletes, and investigate the mechanisms at play. It is possible that the menstrual cycles of female athletes play a role in their perception of sleep and stress. By including gender in the multiple hierarchical linear regression, we attempted to control for these effects. However, future studies should explore the influence of the menstrual cycle on sleep and perceived stress of female athletes on a day-to-day basis.

In non-athletic adults and adolescents, a positive association between psychological resilience and sleep quality is found (Notario-Pacheco et al., 2011; Brand et al., 2016). However, prior to this study, the unique contribution of the different sub-components of mental resilience in sleep quality of junior athletes has been unexplored. In the present study, the mental resilience sub-components Social Resources and Structured Style were positively associated with sleep quality. In fact, of all investigated predictors, the factor Social Resources had the largest unique contribution to sleep quality in the hierarchical regression analysis. The social resources available to junior athletes seem to play an important role in junior athletes’ psychological functioning. Kristiansen and Roberts (2010) have shown that for junior elite athletes, social support is crucial when coping with competitive and organizational stressors, and a supportive coach was found to predict good psychosocial outcomes, which included the absence of achievement-related worries (Ommundsen et al., 2006). Another study showed that the risk of injuries was 70% greater in athletes with high perceived stress, and that the risk for such injuries was predicted by having fewer social resources to manage stress (Steffen et al., 2009). Taken together with the results of the present study, social resources seem to be crucial in protecting against the deterioration of sleep quality in times of stress or adversity.

The mental resilience factor Structured Style represents a measure of personal competence, and refers to the extent an individual engages in planning and structuring daily routines, indicating a preference for approaching matters in a structured way, formulating plans, establishing rules and routines, and being organized toward goal achievement (Friborg et al., 2005). Personal competence is thought to be a crucial aspect of a well-functioning athletic identity, which has now been shown to protect against the worsening of sleep quality in junior athletes. It is suggested that one of the ways in which the factor Structured Style may protect against poor sleep quality is its role in keeping good sleep hygiene (e.g., regular wake and sleep times, limiting afternoon napping and exposure to blue light in the evening hours, avoiding ingestion of caffeine and alcohol, and keeping a healthy sleep environment) which encompasses behaviors and practices that promote continuous and effective sleep. Implementing these routines into the everyday life may be easier for athletes who have a well-developed personal competence, and the ability to plan and structure daily routines. Indeed, recent research has established that in both athletes and non-athletes, poor sleep hygiene is also associated with poor sleep quality (Mastin et al., 2006; Knufinke et al., 2018). The factor Structured Style seems to have a significant resemblance with the trait Conscientiousness (e.g., being organized and prompt) which has been shown to be positively associated with good sleep (Duggan et al., 2014). Hence, the mental resilience factor Structured Style may protect against poor sleep quality in junior athletes.

Further, it was established that worry, but not negative affect, uniquely predicted poor sleep quality in the current sample of junior athletes. In the athletic context, cognitive reactions to stress have mostly been investigated in relation to sleep prior to competitions (Juliff et al., 2015). A recent systematic review (Gupta et al., 2017) on the topic suggested that one possible mechanism involves difficulties with repressing cognitive activity while attempting to sleep (Harvey, 2000). Additionally, the contribution of cognitive reactions to stress in terms of poor sleep quality, sleep disturbances and insomnia has previously been well-established in various non-athletic populations (Harvey, 2002; Kelly, 2002). Importantly, the dominance analysis identified worry to be the second most important predictor of sleep quality, preceded only by perceived stress. One of the possible mechanisms causing these effects may involve deficient modulation of emotional brain responses to aversive stimulation by over-reactivity in amygdala implicated in insufficient sleep (Yoo et al., 2007).

The results in the present study showed that negative affect did not significantly predict sleep quality. This may possibly be due to the relatively high correlation coefficients between worry and negative affect (*r* = 0.47), although the variance inflation factor did not indicate any multicollinearity. Alternatively, these results suggest that sleep quality of junior athletes is more vulnerable to cognitive reactions to stress, despite earlier research showing that negative affect relates to the major psycho-physiological stress loads junior athletes are continuously exposed to Crocker and Graham (1995) and evidence implicating negative affect in poor sleep quality (for a review, see Baglioni et al., 2010). It is not immediately clear why negative affect did not predict sleep quality in the current study, and future research should further investigate this relationship.

Previous research has shown that athletes are exposed to high psycho-physiological stress loads inherent to athleticism (Hausswirth et al., 2014), which may have a detrimental effect on sleep and performance of athletes (O’Donnell et al., 2018b). In the last step of the multiple hierarchical regression, perceived stress was identified as a significant predictor of sleep quality. However, the unique explained variance of perceived stress was 2%. Dominance analysis was subsequently used to identify the predictor of highest relative importance in the model, and showed perceived stress to be the most important. Certain factors should be kept in mind when interpreting these undoubtedly important results. The low unique predictive value of perceived stress is most probably due to the inter-correlations this variable has with the other predictors in the regression model. Still, the predictors together explained 28% of the total variance, which is regarded of moderate strength (Ferguson, 2009).

Importantly, the current findings may potentially extend its implications onto athletic performance as it has been shown that psychological distress affects athletic performance by narrowing athletes’ attention and increasing self-consciousness, injury risk and contributes to discontinuity or even dropout from training (Sabato et al., 2016). Future research should investigate this possibility, and include ecological and temporally relevant measures of stress, athletic performance and sleep quality.

The hierarchical multiple linear regression model used in this study identified the female gender, the mental resilience factors Structured Style and Social Resources, worry and perceived stress as unique predictors of sleep quality. When interpreting the results of the current study, it is important to consider the existing literature showing bidirectionality between sleep quality and stress (Kahn et al., 2013). Previous research has also shown that variations in sleep duration also lead to variations in mood and emotion regulation. Multiple studies have now shown that sleep of either reduced quality or quantity leads to poorer mood and impaired emotional regulation (Zohar et al., 2005; Baum et al., 2014). The role of emotional brain networks and REM sleep seem to be central in the mechanisms that underlie these links (for a review, see Kahn et al., 2013). To consider these complex and bidirectional relationships, which the authors were not able to address in this study due to the study design, future research should employ temporal investigations of how cognitive reactions to stress and perceived stress influence sleep, and vice versa. Utilizing ecological, day-to-day measurements of these variables will allow for thorough investigation of the complex relationships between these variables, for which studies utilizing cross-sectional research, as the present study, only provide a starting point. The present study focused on the notion that negative reactions to stress and perceived stress may worsen sleep quality, and may represent the starting point for future, more thorough research into this largely neglected area of sport psychology.

## Limitations

Regarding the existing literature, the results of the current study are the first to show the unique predictive value of female sex, the mental resilience factors Social Resources and Structured Style, cognitive reactions to stress represented by worry, and perceived stress, on the sleep quality in a large sample of junior athletes. The study was sufficiently powered, as shown by the *a priori* and the *post hoc* power analyses. These findings have important practical and theoretical implications, still they should be interpreted with some limitations in mind. First of all, the response rate in the present study was 34.9%, which poses a risk of potentially producing a bias due to non-response error, in the gathered data. We have no demographic or other data for those who did not participate precluding us from drawing conclusions about non-participants and a possible sample bias the present study. However, in developed, high-income countries, response rates in cross-sectional research utilizing surveys for data collection have been on a steady decline (reviewed in Rindfuss et al. (2015), and thus the relatively low response rate seen in the present study seems to be in line with typical compliance with survey research today.

Furthermore, the results of the current study are limited by the study’s cross-sectional design, and the use of self-report measurements. The cross-sectional design prevents elucidating directionality or causality between study variables. Thus, future research should move from cross-sectional designs in order to address the temporal relationships between the investigated variables, using sleep diaries or ecological and objective methods of sleep measurement, such as actigraphy, and longitudinal study designs. In this way, the bidirectional and/or cyclical nature of the relationships between stress and sleep could be uncovered in athletic samples.

Since all variables were assessed at the same point in time and were self-reported, this poses a risk for the results to be influenced by the common method bias, implying inflated relationships between the study variables (Podsakoff et al., 2003). Lastly, self-report measurements may be subject to recall bias, or inaccurate perception of the posed questions. Future research would therefore benefit from implementing objective measures of stress as well as sleep, which would eliminate some of the limitations present in this study. Hence, the present results should be applied to the population of junior athletes with caution, bearing the limitations in mind.

## Conclusion

To conclude, it is important to point out that even though insufficient sleep quality in athletic populations is well documented, very little research has been dedicated to uncovering the reasons for the inability of athletes to obtain sleep of adequate quality. Based on the results of the present study, it is clear that close attention should be paid to athletes’ abilities to manage worry and perceived stress, consequential to the inherent psycho-physiological stress loads of athleticism, in order to prevent poor sleep. This might be especially relevant for female athletes. Not only has poor sleep been firmly implicated in the decline of various performance parameters, it may also be a sign that the athletes may not have the necessary social resources and sense of personal competence needed to balance their psycho-physiological stress loads, and ultimately, progress in their sport. Since performance margins are progressively becoming smaller and smaller, every improvement that adequate sleep can provide will be beneficial to the progress in performance. Therefore, the results presented in this study concerning how sex, cognitive reactions to stress and perceived stress are uniquely associated with sleep quality, and how mental resilience may provide a protective function in this relationship, is of crucial importance for the arena of athletic research and practice. For coaches, the association between athletes’ sleep quality and the psychological variables provide important knowledge, giving the coaches an option to intervene or offer support to athletes obtaining insufficient sleep quantity or quality when necessary. Future research should investigate the effects of strategies to improve mental resilience in junior athletes, such as mindfulness, cognitive-behavioral therapy, or biofeedback training, in order to alleviate the burden of stress and improve sleep quality.

## Data Availability

The datasets generated for this study are available on request to the corresponding author.

## Ethics Statement

This study was carried out in accordance with the recommendations of REC, the Regional Committees for Medical and Health Research Ethics in Norway, with written informed consent from all subjects. All subjects gave written informed consent in accordance with the Declaration of Helsinki. The protocol was approved by REC.

## Author Contributions

MH and FM contributed to the conception and design of the study, collected the data, and carried out statistical analyses. MH wrote the manuscript. FM supervised the data collection and preparation of the manuscript. SP assisted with statistical analyses and edited the manuscript. All authors were involved in the interpretation of the statistical analyses and approved the final version of the manuscript.

## Conflict of Interest Statement

The authors declare that the research was conducted in the absence of any commercial or financial relationships that could be construed as a potential conflict of interest.
