# Supplementary material for: Unique Predictors of Sleep Quality in Junior Athletes: The Protective Function of Mental Resilience, and the Detrimental Impact of Sex, Worry and Perceived Stress
Source: Front Psychol. 2019 May 31;10:1256. doi: 10.3389/fpsyg.2019.01256 (PMC6554288; doi:10.3389/fpsyg.2019.01256)
Supplement: Supplementary file 1 [file Table_1.docx]

Supplementary Material

**1 Results of assumption tests for partial correlation statistics**

1.1 Normality

The Kolmogorov-Smirnov and Shapiro-Wilk tests of normality and tests of skewness and kurtosis were significant for all investigated variables. However, it is not recommended to run normality tests on large sample sizes, as is the case in this study (*N*=632) (Field, 2009, page 139, 148). Therefore, we also visually investigated the histograms for each of the continuous variables investigated in this study, which supported the non-normality results in most variables. Based on these results, Spearman’s rho correlations were applied.

1.2 Variable types

All independent variables (apart from sex) as well as the dependent variable were measured on a continuous scale. Sex was the only categorical variable, and used as a control in the Spearman’s rho correlations.

1.3 Linearity

Based on simple scatter plots with fit lines, created in SPSS graph builder, all variables had linear relationships with sleep quality as measured with PSQI.

1.4 Outliers

There were no significant outliers.

**2** **Results of assumption tests for multiple regression statistics**

2.1 Normality of distributed errors

For the multiple regression analyses, the normality assumption applies to the error distributions (residuals), rather the predictors and the outcome variables (Field, 2009, page 221). This assumption has been checked using the Predicted Probability (P-P plot) in SPSS. No drastic deviations from the line (i.e. points crossing the line horizontally) were identified, which implies no drastic deviations in the distribution of errors.

2.2 Multicollinearity

SPSS has been used to produce the variance inflation factor (VIF), a measure of multicollinearity, which indicates the strength of the linear relationship between predictors. Two indicators may be used to evaluate whether there is cause for concern in regards to multicollinearity. Firstly, values that are greater than 10, and secondly, when the average of all VIF factors is much greater than 1 (Field, 2009, page 242). Neither of these indicators are present in the data (see Table 1 for the VIF values in Model 1, and Table 2 for VIF values in Model 2).

| Table 1 *Multicollinearity diagnostics for multiple linear regression investigating predictors of sleep quality, Model 1, based on cross-sectional data in 632 junior athletes.* | |
| --- | --- |
| Variable | VIF |
| Sex | 1.24 |
| Perception of self | 2.74 |
| Planned future | 1.96 |
| Social competence | 1.37 |
| Family cohesion | 1.86 |
| Social resources | 2.26 |
| Structured style | 1.42 |
| Negative affect | 1.64 |
| Worry | 2.19 |
| Perceived stress | 2.97 |

| Table 2 *Multicollinearity diagnostics for multiple linear regression investigating predictors of sleep quality, Model 2, based on cross-sectional data in 632 junior athletes.* | |
| --- | --- |
| Variable | VIF |
| Model 1 |  |
| Sex (♂=0, ♀=1) | 1.00 |
| Model 2 |  |
| Sex (♂=0, ♀=1) | 1.01 |
| Social resources | 1.01 |
| Model 3 |  |
| Sex (♂=0, ♀=1) | 1.03 |
| Social resources | 1.14 |
| Structured style | 1.17 |
| Model 4 |  |
| Sex (♂=0, ♀=1) | 1.17 |
| Social resources | 1.22 |
| Structured style | 1.17 |
| Worry | 1.21 |
| Model 5 |  |
| Sex (♂=0, ♀=1) | 1.20 |
| Social resources | 1.34 |
| Structured style | 1.27 |
| Worry | 1.93 |
| Perceived stress | 2.30 |

2.3 Homoscedasticity

Scatterplots of residuals have been examined to check homoscedasticity. The data points were distributed above and below zero on the X axis, and above and below zero on the Y axis, implying the data is homoscedastic.

2.4 Linearity

Based on simple scatter plots with fit lines, created in SPSS graph builder, all variables have linear relationships with sleep quality as measured with PSQI.

2.5 Variable types

All predictor variables were quantitative and continuous, with the exception of sex, which was a categorical variable with two categories. The outcome variable was quantitative, continuous and unbounded.

2.6 Non-zero variance

The predictors did not have variances of 0.

2.7 Independent errors

The Durbin-Watson test was carried out to check for autocorrelation between the investigated variables. The values of the Durbin-Watson test were 2.14 and 2.16 for Model 1 and Model 2, respectively. These values lie between the critical cutoff of 1.5 < d < 2.5, which gives reason to assume that there is no first order linear auto-correlation in our multiple linear regression data.

2.8 Independence

Each value of the outcome variable comes from a separate entity.

**2 Results of the multiple linear regression testing Model 1**

| Table 3 *Multiple linear regression analysis for variables predicting subjective sleep quality, based on cross-sectional data collected from 632 junior athletes.* | | | | | |
| --- | --- | --- | --- | --- | --- |
| Predictor | *B* | *SE B* | β | *t* | *p* |
| Sex (♂=0, ♀=1) | .45 | .23 | .08 | 2.00 | .047 |
| Perception of self | -.06 | .15 | -.02 | -.41 | .679 |
| Planned future | -.11 | .11 | -.05 | -.97 | .331 |
| Social competence | .19 | .11 | .07 | 1.72 | .087 |
| Family cohesion | -.14 | .14 | -.05 | -.10 | .320 |
| Social resources | -.44 | .18 | -.13 | -2.47 | .014 |
| Structured style | -.38 | .10 | -.15 | -3.74 | .000 |
| Negative affect | .03 | .02 | .07 | 1.68 | .093 |
| Worry | .04 | .01 | .17 | 3.33 | .001 |
| Perceived stress | .06 | .02 | .15 | 2.53 | .012 |
| R^2^ | .28 | | | | |

**3 References**

Field, A. (2009). *Discovering statistics using SPSS.* Sage publications.
